# Citizen Consultation on Problematic Usage of the Internet: Ethical Considerations and Empirical Insights From Six Countries

**DOI:** 10.3389/fpubh.2021.587459

**Published:** 2021-04-01

**Authors:** Biljana Gjoneska, Julia Jones, Anna Maria Vella, Philip Bonanno, Katerina Flora, Andrés Fontalba-Navas, Natalie Hall, Liljana Ignjatova, Zviad Kirtava, Daniel Moreno Sanjuán, Maria Piedade Vaz-Rebelo, Célia M. D. Sales

**Affiliations:** ^1^Macedonian Academy of Sciences and Arts, Skopje, Macedonia; ^2^Centre for Research in Public Health and Community Care, University of Hertfordshire, Hatfield, United Kingdom; ^3^Faculty of Medicine and Surgery, University of Malta, Msida, Malta; ^4^Faculty of Education, University of Malta, Msida, Malta; ^5^Psychology Department, Neapolis University, Pafos, Cyprus; ^6^Antequera Hospital, Northern Málaga Integrated Healthcare Area, Andalusian Health Service, Antequera, Spain; ^7^Department of Public Health and Psychiatry, University of Malaga, Malaga, Spain; ^8^Centre for Health Services and Clinical Research, University of Hertfordshire, Hatfield, United Kingdom; ^9^Faculty of Medicine, Ss Cyril and Methodius University, Skopje, Macedonia; ^10^Centre for Prevention and Treatment of Drug and Other Substance Dependence, Psychiatric University Hospital, Skopje, Macedonia; ^11^Department of Medicine, Tbilisi State Medical University, Tbilisi, Georgia; ^12^Nongovernmental Research Organization “Partners for Health”, Tbilisi, Georgia; ^13^Andalusian Health Service, Seville, Spain; ^14^Faculty of Psychology and Educational Sciences, University of Coimbra, Coimbra, Portugal; ^15^Faculty of Psychology and Education Sciences, University of Porto, Porto, Portugal; ^16^Centre for Psychology, University of Porto, Porto, Portugal

**Keywords:** ethical considerations, conceptual framework, emerging discipline, early research, public health, problematic usage of the internet, citizen consultation

## Abstract

Citizens and scientists can work together to improve the collective well-being, if citizens are inspired to help the advancement of science, and researchers motivated to listen to the voices of citizens. The benefits of such collaboration are increasingly recognized by both citizens and scientists, as reflected in the growing number of related publications and initiatives. This is especially relevant for emerging areas of research, where early involvement of citizens could help to envision, prioritize, and plan prospective studies. The Problematic Usage of the Internet (PUI) is one such area, which is fast becoming a public mental health concern. However, there remains a lack of clarity regarding the practical guidelines and ethical requirements for citizen involvement at the earliest stages of PUI. In our paper, we propose a conceptual framework and a template for initial involvement of citizens in PUI. They are derived from our community case studies, conducted in six European countries (Georgia, Greece, Malta, North Macedonia, Portugal, and Spain) and consisting of consultation with diverse groups of interested citizens (students, parents, teachers, and health professionals). Informed by our consultation exercises, we also highlight four ethical aspects for citizen involvement in the research on PUI or novel disciplines in general. They follow simple guiding principles to ensure that scientists will: enable a long-term commitment and inclusive opportunities for citizens, challenge established power hierarchies, and support collaboration, co-production and co-authorship with citizens. We believe that the proposed practical guidelines and ethical considerations, provide a valuable foundation on which to advance our understanding and generate international strategies for citizen involvement in PUI.

## 1. Introduction

Citizens from all walks of life and researchers in diverse fields of science often share an interest in a particular topic with great potential for collaboration. Citizen involvement in research, is one such collaboration in which a beneficial and reciprocal partnership may be developed. This collaboration could inspire citizens to gain enhanced understanding of the scientific matters and to help researchers in the advancement of science. In return, it could also motivate researchers to listen and value the views of citizens, but also involve them in future research. The benefits derived from inviting members of the public to participate in research projects are becoming increasingly recognized, as reflected in the growing number of related publications and initiatives, from 2003 onward (1). However, there remains a lack of clarity regarding the ethical requirements for citizen involvement in research, particularly at the early development stage of research. In this paper, we summarize our experiences from citizen consultation in the novel area of the Problematic Usage of the Internet (PUI).

This collaborative approach spans areas like Patient and Public Involvement (PPI) and Citizen Science, underpinned by the principle that the research should be “carried out *with* or *by* members of the public [and patients] rather than *to, about* or *for* them” (2). There is ample evidence on the enhanced quality of health research, with a plethora of models, frameworks, guidelines and toolkits produced to guide future strategies for citizen involvement (3–5). The potential for new conceptualizations of PPI in research, becomes especially relevant in the case of emerging research areas, which can present unique characteristics and merit special attention. Novel disciplines are fast advancing yet still developing, hence many questions are still unresolved and open to interpretations or re-conceptualizations. In these circumstances, citizens can play an important role and provide useful insights for specific research questions, but also contribute to the broader field in general. This is the case, for example, with as-yet untapped potential of PPI to assist research into PUI. The present study focuses on consultation with *citizens* (i.e., lay people or the general public) rather than *patients* (as is the case of many previous studies conducted in health research), and our *citizen consultation exercises* represent an innovative way of working in the emerging field of PUI. We will propose that the early involvement of citizens in PUI can help to envision, identify, prioritize and plan future research in this emerging research area, which has relevance to citizens and researchers alike (6).

Problematic Usage of the Internet is an umbrella term referring to a range of possibly harmful internet-related activities, including “video gaming, gambling, buying, pornography viewing, social networking, ‘cyber-bullying' and ‘cyberchondria' among others” (7). It is relatively novel area, but a topic of international public mental health concern, recognized as such by the World Health Organization (8), international research collaborative networks (7) and national governments (9).

To the best of our knowledge, no prior studies have been conducted to address the ethical considerations regarding the future involvement of citizens in research on PUI. Indeed, there is a paucity of research and guidance *per se* regarding research ethics and ethical requirements for citizen involvement at planning stage in any emerging research area. In UK however, there is guidance from the INVOLVE initiative, that was until recently part of the National Institute of Health Research (NIHR). According to INVOLVE guidance from 2016, ethical approval is not needed “to involve the public in the planning or the design stage of research” (10). This guidance clearly distinguishes between members of the public who are collaborators in the research process, compared to being research participants. However, it remains important that an ethically conscious and cautious approach should be exercised when involving citizens in the research process, as outlined by Pandya-Wood et al. (11), so as to ensure that the moral principles are maintained throughout the research process, thus protecting the rights, safety, dignity, and well-being of all parties.

In this paper, we propose theoretical and practical insight for citizen involvement in research on PUI, to enrich the area of public mental health. Specifically, we present: (a) a conceptual framework for early engagement of citizens in emerging research, specifically at the planning research stage; and (b) a template for initial consultation with citizens on PUI. To address these aims, we present community case studies of citizen consultation conducted in six European countries between April-August 2019. These consultations were conducted as part of our citizen involvement activities within the European Network for Problematic Usage of the Internet (EU COST PUI) (12). We conducted the consultation exercises to listen to the views, concerns and experiences of different groups of citizens about PUI, in order to inform the direction of our future research. It is important to note that the information collected throughout the consultation exercises was not treated as data, nor subjected to qualitative analysis. We have used the information gained from these sessions, to develop practical guidelines and ethical considerations for the early involvement of citizens in PUI.

## 2. Research Context

**Figure 1:**
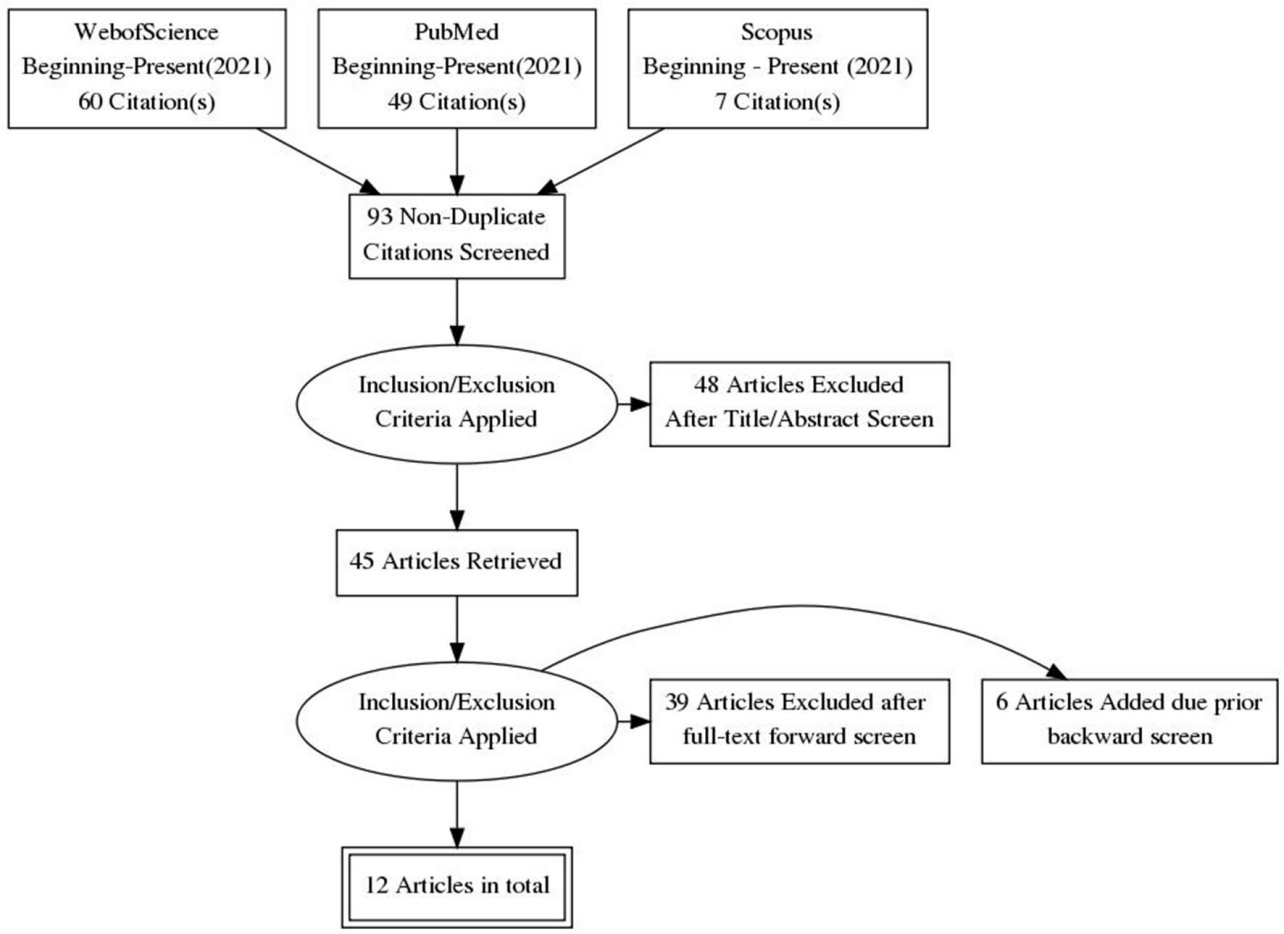
The diagram depicts the process of selecting relevant studies that consider the ethical aspects of citizens involvement in planning stages of research. It is prepared in accordance with the PRISMA standards for selection of studies and reporting of items for systematic reviews and meta-analyses. The targeted search through comprehensive databases (Web of Science, Scopus, and PubMed), was followed by screening of retrieved studies, and concluded with final selection of relevant articles.

**Figure 2:**
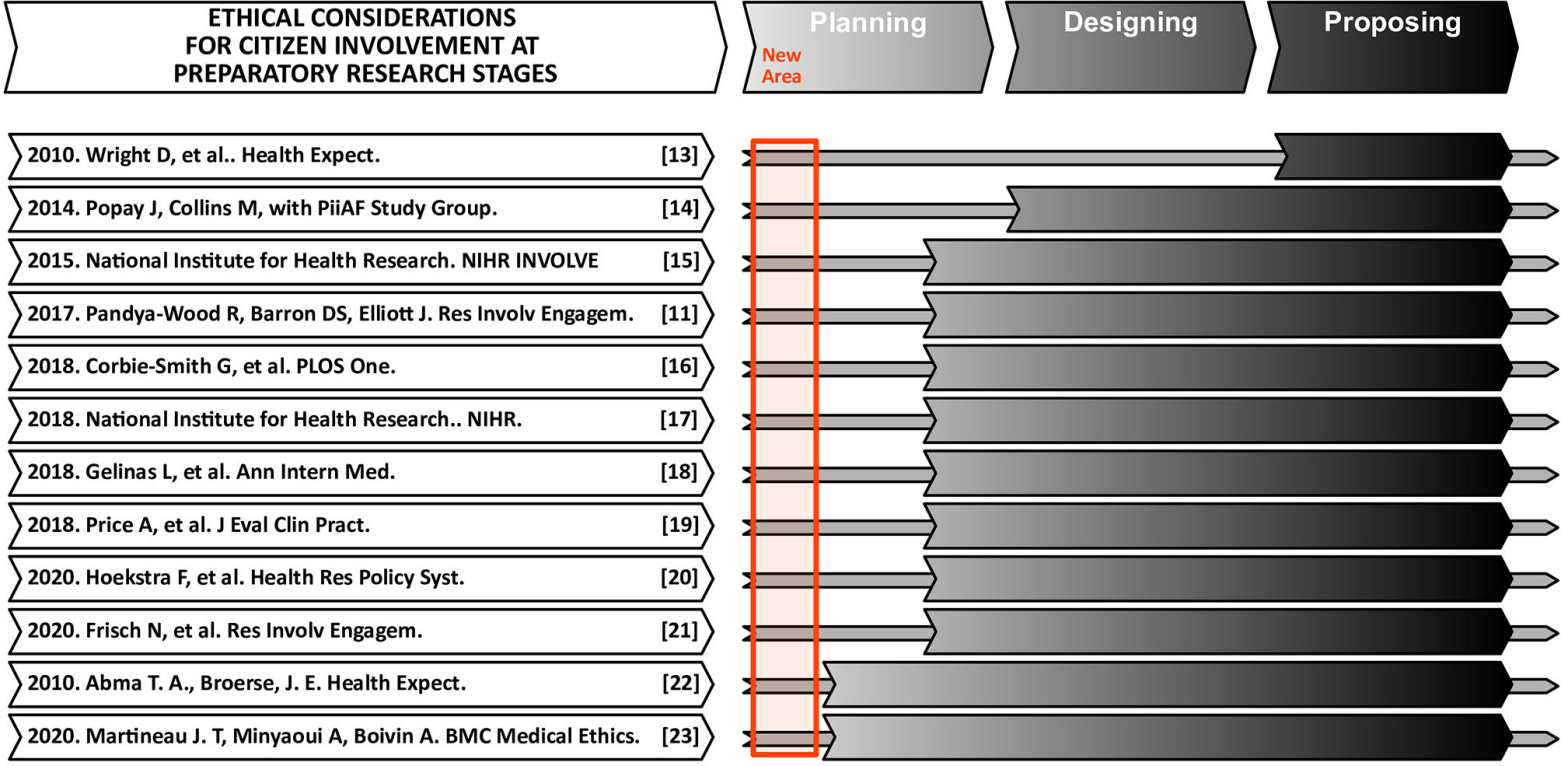
The illustration lists a selection of 12 relevant publications, that directly or indirectly consider the ethical aspects of citizens involvement in research. Specifically, the list includes papers that discuss citizen involvement at preparatory stages of the research along a chronological axis (from planning, design, and submission, to approval of proposals). The involvement of citizens in executive stages of the research (conducting and implementing a project, or dissemination of results) is not addressed in the list. As evident, none of the selected studies have considered the early involvement of citizens in new and emerging areas of science (like PUI).

**Figure 3:**
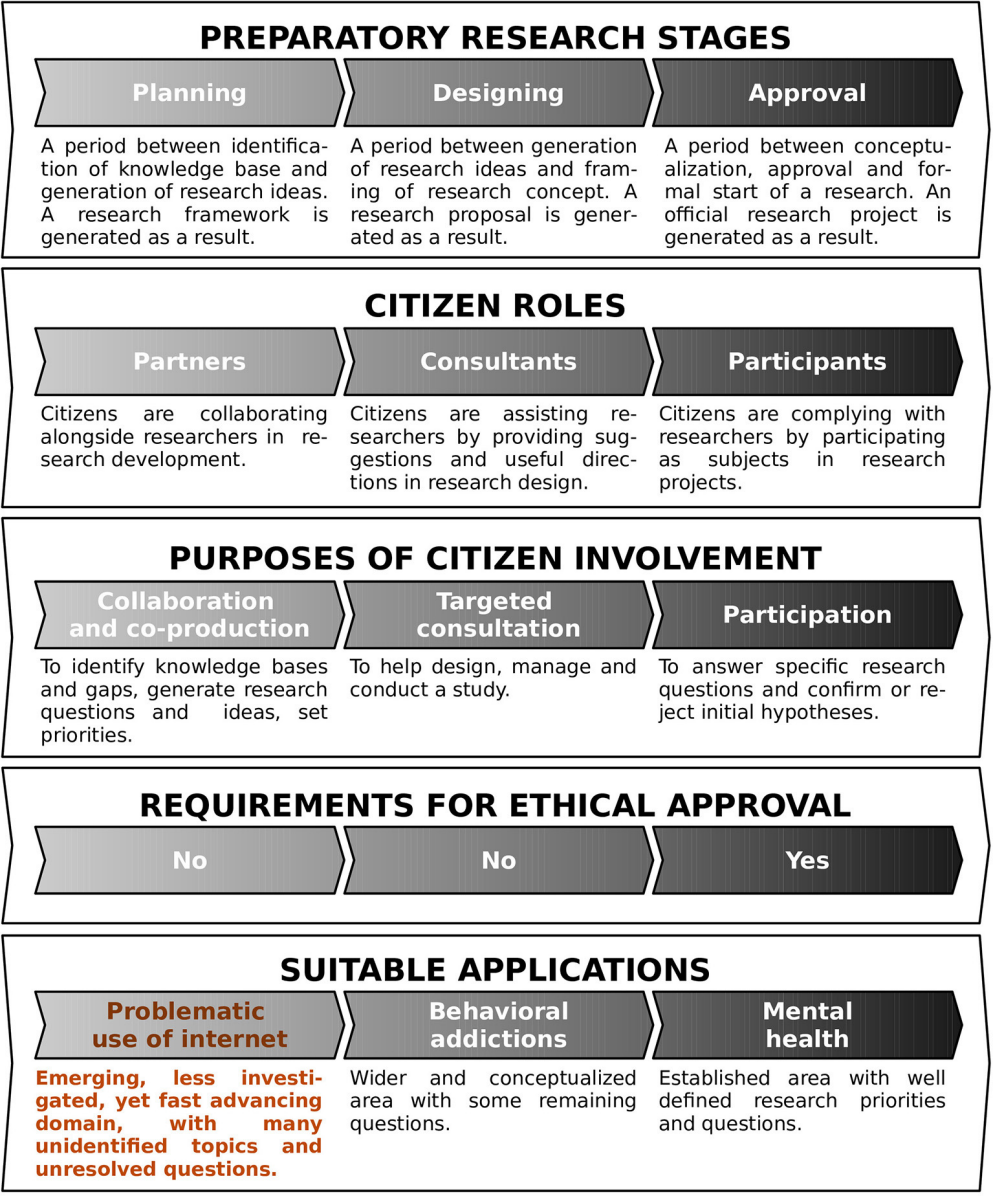
The illustration outlines the main aspects of citizen involvement at the earliest stages of a research project, with special emphasis on their involvement in emerging areas of science. It highlights the roles played (as research partners, assistants, or participants), the nature of the commitment (based on co-creation, consultation, or participation), and the ethical requirements involved (where, depending on the stage of research, citizens may be consulted, or they may constitute the object of the study).

The theoretical part of this paper builds upon prior literature, which informed our approach for the consultation exercises. For this purpose, we first conducted broad searches (in the title, abstract, and keywords of potential manuscripts), across comprehensive databases (Web of Science, Scopus, and PubMed), related to our specific topic of interest (by combining keywords on “citizen / public/ patients/ people” with “consultation / involvement / engagement” about “internet / cyber / online / virtual” and “problem / addiction / obsession”). As expected, the search returned zero or handful of results. Hence, we utilized a more general strategy to tackle the broader research context (Figure 1 presents a diagram of the selection process). Publications were deemed relevant if they adopted the following inclusion criteria: (a) as regards the scope, they consisted of reviews and meta analyses; (b) as regards the topic, they focused on ethical aspects for citizen involvement in planning stages of research processes. On the other hand, the articles were considered for exclusion if they comprised case studies and were restricted to investigations in specific research area, population or country. The final selection included frameworks, guidelines, recommendations, handbooks, and checklists, but most importantly of reviews, reviews of reviews, and even frameworks of frameworks (see Figure 2) (11, 13–23). A relatively small selection of publications considered the ethical aspects for citizen participation in research processes, in step-by-step fashion (see Figure 2). As can be seen, the list focuses on citizen involvement at preparatory stages (planning, designing, submitting, or approving proposals), and does not address executive stages of the research (conducting research or disseminating results). For example, Pandya-Wood et al. (11), clearly identify the need to adopt an ethically conscious approach even prior to the formal approval of a study, and discuss the main ethical issues encountered in the design stage. However, all these authors fundamentally consider citizen involvement within the life-cycle of a specific project, and do not address the broader scope of a research discipline. The other authors (13–16, 24), mention the need to involve patients at the “ideas stage” of a study, but rarely discuss the potential perspectives for involvement of citizens in relatively novel disciplines. In short, the earliest degree of citizen involvement, before the actual study design takes place, or even before a discipline is officially established, has received virtually no research attention from an ethical standpoint. As outlined on Figure 3, citizens can help scientists grasp the magnitude of the problem, envision future research, map research priorities, identify key topics or hot issues, generate research questions, and plan future studies. Besides highlighting potential role of citizens as research partners, the framework we propose outlines the nature of their commitment (based on collaboration, co-creation and co-production) and clarifies the lack of need for written consent or ethical approval (based on the fact that citizens are consulted in a study, rather than subjected to a study).

## 3. Citizen Consultation Exercises on PUI

Citizen consultations were planned and conducted over a 5-month period and across six countries, located in southern Europe: Georgia, Greece, Malta, North Macedonia, Portugal, and Spain (listed in alphabetical order). The participating countries were selected according to the following inclusion criteria: (a) they were all countries represented by members of the Working Group for Citizen Involvement (WG5) within the European Network for Problematic Usage of the Internet (EU COST PUI); (b) all countries were without national strategies or guidelines for PPI in PUI, or health research in general (25). In preparation to undertake this international citizen consultation, PPI training was provided to the lead author (BG) and members of the WG5, by the second author (JJ) who has expertise in PPI and Citizen Involvement and leads an academic PPI research group at a UK University. The wider WG5 membership was coordinated to plan for the consultation exercises, via series of live and virtual meetings, email and organizational platforms. It was agreed to include citizens with the following characteristics: (a) adults (depending on the country, the citizens were at least 18 or 21 years and older); (b) had direct or indirect contact with the topic of PUI as students, parents, teachers or health professionals. Then the WG5 members in their respective countries made contact with groups of citizens, who met these criteria, and invited them to participate in a consultation meeting.

Thirteen groups of citizens were consulted between April and August 2019. The average group consisted of 15 people, with variations in size between 5 (Georgia) and 27 (Malta) individuals, encompassing a total of 194 citizens (across all countries). Different groups of citizens were involved in the different countries: Spain and Georgia organized consultation with parents (mothers), while Greece consulted with mixed groups of parents (mothers and fathers). Teachers were consulted in Malta and North Macedonia, while students (aged above 18 years) were consulted in North Macedonia, Portugal, and Georgia. Additionally, in North Macedonia consultation groups were also conducted with health professionals (nurses, physicians, social workers). All citizens were informed about the nature and the scope of the exercise and verbally consented to participate in the consultation on PUI.

In all countries, the consultation exercises were carried out in a single session, either face-to-face or online. The face-to-face meetings were conducted in professional surroundings (such as universities, hospitals or conference centers). The settings were familiar to the citizens, the atmosphere informal, and the consultation was conducted in a conversational manner as a semi-structured interview. In each country, the exercise was coordinated by national COST Action representatives, who were experienced researchers. They had existing relationships and ongoing collaborations with the groups, or prior contacts with some of the citizens. The discursive and open nature of the discussions fostered spontaneity and empowered the participants to engage freely. As a result, the responses were motivated and uninterrupted, and the groups as a whole engaged in fluid, lively discussions.

**Figure 4:**
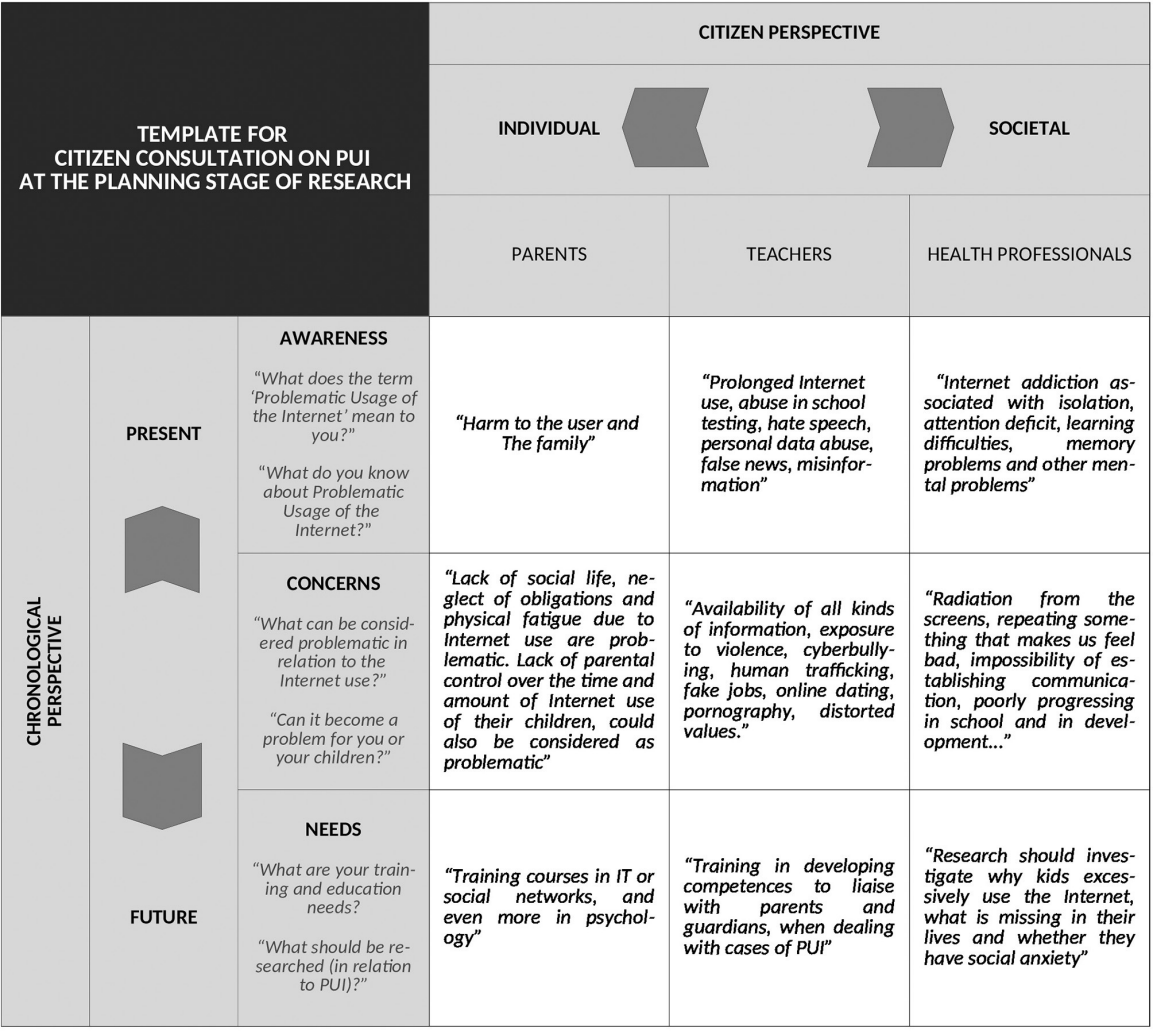
The template illustrates the typical organization of an initial citizen consultation exercise on PUI. The vertical axis covers the chronological perspective, following the sequential order of addressed topics throughout the course of the exercise. The horizontal axis covers the individual perspective of interested groups of citizens. The first perspective expresses the present awareness, concerns and future needs of consulted citizens, while the second perspective describes their fixed opinions. The template also includes an illustrative list of the questions posed by the researchers, and a selection of sample quotes from some of the consulted citizens.

The discussions in the consultation exercises were guided by a shared topic guide that was developed in advance by an interdisciplinary group of researchers and practitioners and WG5 members. The idea was to formulate broad open-ended questions, that would stimulate citizens to freely express opinions and start a conversation. The list could be adjusted by the national facilitator to ensure that the questions were relevant and understandable for the national and local context, as well as for the specific group of citizens. See Figure 4 for the topic guide used for the consultation exercise. For instance, when discussing the prior knowledge on the subject of PUI, the researchers posed several iterations of a similar question: “*What does the term ‘Problematic Usage of the Internet' mean to you?*”, “*In your opinion, what is Problematic Usage of the Internet?*”, “*What can be considered as problematic in relation to the Internet use?*” and “*What do you know about Problematic Usage of Internet?*” In this way, the researches were able to adjust to the personality traits, cognitive styles or worldviews of the citizens consulted. In consequence, these consultation exercises acquired some beneficial properties of group sessions (providing supportive and understanding environment for all involved parties), whilst extracting sincere, engaged and relevant contributions from the group members.

In general, the exercise was organized in such a way, as to take into account both perspectives: the individual (following the thought processes of the citizens consulted) and the chronological perspective (following the course of the exercise). The scheme is illustrated in Figure 4. Regarding the chronological perspective, the session was first focused on the citizens' awareness of PUI issues, and considered their future needs at a later point. Discussion of potential *concerns*, provided a transition between the discussions on the *present awareness* and the *future needs* of citizens. When considering citizens' awareness, researchers sought insights into the knowledge base and knowledge gaps of citizens (i.e., what citizens think they know and what they really knew about the problem). In other words, the researchers wished to identify the participants' subjective and objective understanding of the questions raised. In addressing potential concerns, the researchers were also informed of the citizens' attitudes, beliefs, difficulties and specific worries associated with the problem. Finally, the discussions of future needs, revolved around the pressing questions that need to be tackled by research on PUI.

Overall, the researchers from the six European countries were encouraged by the level of interest in the topic of PUI and the citizen participation in the initial consultation exercises. There was a consensus among researchers that the level of interest from citizens in different countries was a promising sign for their future consultation and inclusion in the development of national guidelines for PPI on PUI. As regards to specific experiences, two general impressions were formed, based on researchers' notes and summaries from the consultation exercises. Those researchers who conducted consultation with *parents* have consistently reported parents' *concerns and needs related to PUI* that were repeatedly voiced across multiple consultation settings: (a) a concern about Internet usage in children; and (b) a need for training and education about PUI of parents themselves. Whereas, the researchers who consulted *teachers and health professionals* have noted the professionals' *awareness about PUI*, regardless of the consulted group or country. In fact the professionals' knowledge base (in terms of definitions and descriptions of PUI) and their identification of knowledge gaps (in terms of reported weaknesses and shortcomings) were quite rich, observant and insightful. Nevertheless, it appeared that many *professionals* from the different countries have sought to improve their personal skills and competencies around PUI. Whilst the *parents* focused on the need for psychological advice and counseling in support of their children or families.

## 4. Discussion of Key Ethical Aspects for Future Involvement of Citizens in PUI

**Figure 5:**
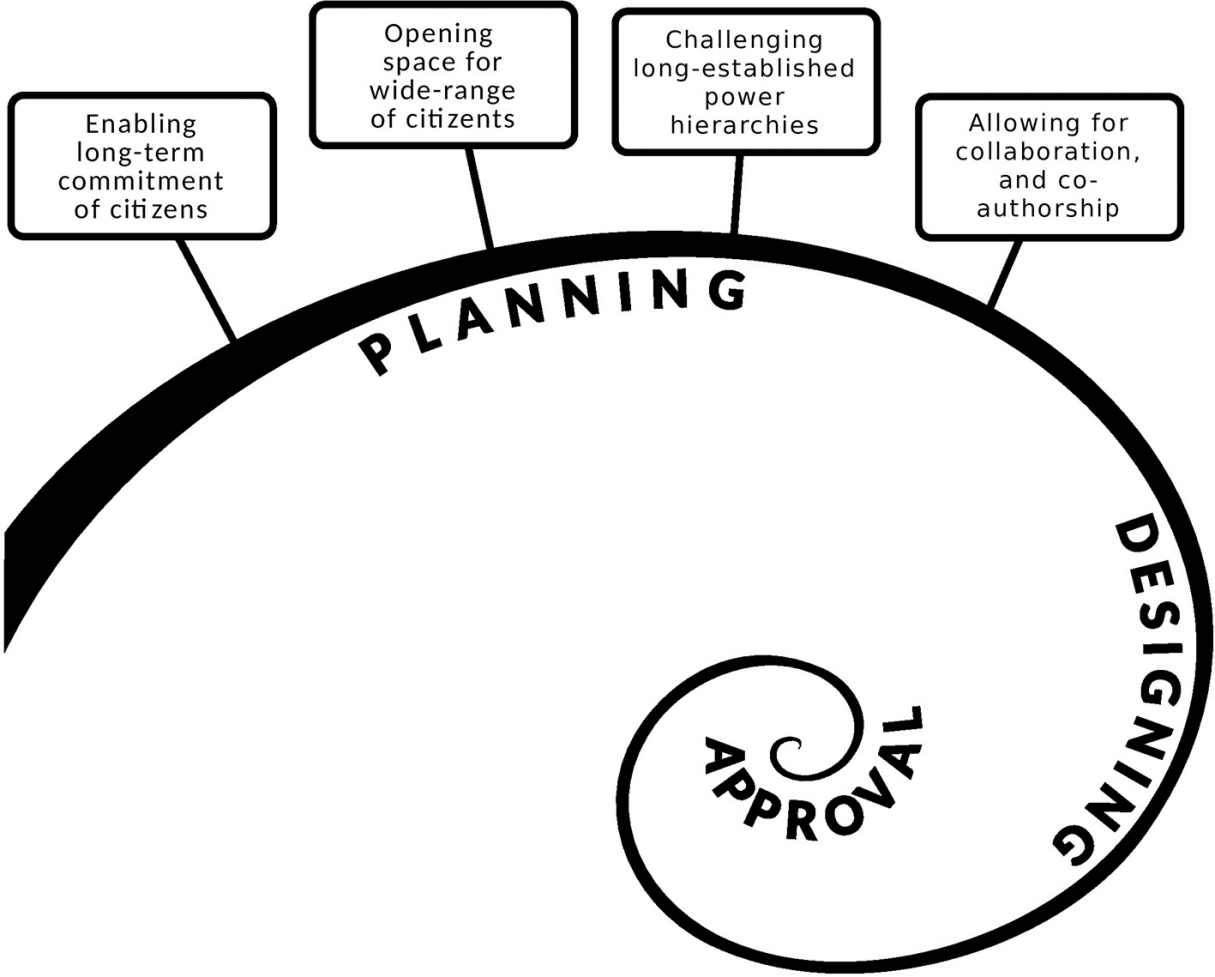
The illustration highlights the main ethical considerations underlying the inclusion of citizens at the planning phase of PUI. As can be seen, the planning phase in emerging research areas (which precedes the project design, submission, and approval), is the most time consuming. Hence, it should be conducted in a cautious and ethically conscious manner.

In the following section, we focus on main ethical aspects that should be taken in consideration upon future inclusion of citizens in research on PUI, especially in the planning and design stages of research (also shown in Figure 5). They were identified from our community case studies, conducted to ascertain the opinions, concerns and questions of citizens on the subject of PUI.

### 4.1. Enabling the Long-Term Commitment of Citizens

Time-related issues were frequently raised during consultation exercises, corroborating the findings from previous research (11, 26). Consulted citizens highlighted the need for time to acquire sufficient experience with Internet technologies and with the specific research on PUI, in order to become involved in a meaningful way at the early stages of research development. In addition, the discussion groups have addressed the time-sensitive issue of maintaining motivation and sustained dedication, that are required on both sides for the purpose of obtaining significant scientific outcomes. Therefore, engaging with citizens in sensible, respectful, and equally dedicated manner, would enhance the probability of achieving real progress over longer periods. The prospects for long-term and sustained collaboration with citizens on PUI, could be particularly improved by reaching out to existing citizen groups, such as established consumer groups, school parent groups and youth groups. In addition to joint meetings and consultations, the members of these group could also be assigned to training courses, invited to public events and community presentations, as well as continually informed and updated via social platforms on all developments regarding PUI.

### 4.2. Inclusive Opportunities for Involvement

The importance of inclusivity and diversity in PPI (11, 13) was also emphasized in our consultations with citizens on the subject of PUI. The consulted adults expressed a regard that younger generations (children, adolescents, and young people in general) are more vulnerable to PUI, but also more knowledgeable on the subject, so they represent a valuable source population for identifying priorities for future research in this area. This sentiment has been expressed in earlier work on cyberbullying (27). Still, to this date, little research has been conducted as a result of collaborative scientific investigations with different groups of citizens, on the health impact of Internet technologies (as hallmarks of the new age and as relatively recent phenomena). The inclusion of a wide range of citizens with a variety of backgrounds, could help to better understand the magnitude of PUI at different points in time and space, and across various settings and circumstances. Identifying the most relevant groups for involvement in PUI research, becomes particularly important in times of the pandemic, when spatial restrictions and behavioral adaptations can exert great influence over the course of both COVID-19 and PUI among vulnerable populations. In relation to this, the following section offers rationale for usefulness of future consultation with young adults (aged between 18 and 25 years) (28).

### 4.3. Challenging Long-Established Power Hierarchies

The notion of reciprocity was discussed during the consultation exercises, and the need for education on PUI was emphasized. The citizens regarded it as a way to become better informed and empowered, and to contribute on more equal terms in the research. Power imbalances can pose a threat if disproportional and discriminating tendencies emerge in the relationships between scientists and citizens, manifesting as an increased sense of entitlement (among scientists), or an increased distrust in scientists (among citizens), thus hindering all prospects for fruitful collaboration between two sides.

We suggest that the early involvement of citizens in emerging research areas, could challenge existing power imbalances and decrease inequalities (29, 30). By becoming collaborators at an early stage of the research, citizens would establish reciprocal relationships with researchers and their voices would be heard and acted upon. Such a development would be highly significant in the case of PUI, an area in which health professionals are struggling to overcome the power hierarchies of the business sector and break down the silos of the computer, video gaming, and gambling industries (31), to make society aware of potential health risks. We argue that the inclusion of citizens in the early stages of PUI research would strengthen the evidence generated in collaborative research and give a voice to the end-users. In short, it would empower both interest groups to a similar extent, with a wealth of credible, legitimate and shared knowledge.

In this regard, future consultations with advisory groups consisting of young people (aged 18–25), might be an advisable way to proceed and a positive route to address all three concerns: the long-term commitment, the inclusive involvement and empowerment of citizens. This is based on the following reasoning:

(a) The proposed age-group consists of people who are young enough to have had a significant exposure to the Internet, but mature enough to understand the associated risks and possible complications. We are aware that this age group often utilize Internet to satisfy their social and emotional needs, but also for educational and work-related purposes. Hence, they should have *genuine interest on the subject of PUI and be naturally incentivized for long-term collaboration*.

(b) Young people have been growing-up fully immersed in the world of Internet, so they should have considerable knowledge and technical prowess. In fact, we believe that young people *might already be considered as equal partners, when considering the issues of digital literacy and technical Internet skills*.

(c) Most importantly, young people *may have the time and educational interest to invest in long-term engagement* based on collaboration, co-production and co-authorship. Young people who are in education may view such collaboration a good opportunity for their personal learning and development and support the next steps in their career aspirations.

### 4.4. Supporting Collaboration, Co-production, and Co-authorship

Closely related to all of the above aspects of citizen involvement in research on PUI, are the discussions currently taking place in PPI on greater fairness and recognition for citizens, in terms of collaborative and reciprocal relationships, co-production and co-authorship (17, 32). Ideally, the relationship between citizens and scientists will advance with the advancement of the research in the area of PUI. In such circumstances, the level of citizens' expertise might become comparable to that of the scientists, so this might include a move toward co-production, where decisions about the research are shared and everyone's expertise is valued. This includes co-authorship of reports, publications and other scientific outputs (33). Nevertheless, different groups of citizens, can offer unique and useful insights regardless of their level of involvement or expertise, so each contribution should be properly valued and adequately acknowledged.

### 4.5. Potential Limitations

The conceptualization we propose for the early involvement of citizens in the emerging research on PUI, should be considered within the context of existing frameworks and guidelines for PPI in the field of health sciences. These prior efforts provide the background to our call for greater citizen involvement in PUI research. However, as argued by Greenhalgh et al. and by NIHR INVOLVE, the development of a “one-size-fits-all” model seems no longer viable. Instead, the idea of “building one's own framework” is becoming increasingly accepted. This has been useful for us, as we consider how best to involve citizens in future research on PUI. In this respect, too, the UK National Institute for Health Research advises that PPI experiences involving the joint input of citizens and scientists should be documented in academic publications, and thus made available to future generations of researchers (24). We support these recommendations and offer a conceptualization that stems from our consultation exercises, which may prove useful to other researchers working in related areas.

Regarding the limitations of our work, we are well aware that our conclusions are derived from exercises in European countries with limited experience on PPI in research, and (to our knowledge) no known evidence of PPI in PUI (25). With this in mind, we took all possible precautions to compensate for possible methodological constraints. Importantly, the greatest possible consensus was sought among the partners collaborating in this project, so that the proposed conceptual framework and practical guidelines would properly reflect the opinions of all parties.

Finally, it should be noted that the proposed ethical considerations for the early involvement of citizens in the emerging field of PUI do not represent an exhaustive list, but could be extended to include other useful considerations by researchers in related fields (11, 23, 34). Thus, issues such as tokenism (i.e., treating the consultation exercises like “tick-box” exercises), or lack of feedback for involved citizens (i.e., debriefing citizens on the ways their involvement has been taken forward by researchers), are also relevant in the context discussed. For the purpose of this paper, our focus was limited to the ethical issues that arose from our consultation exercises on research in PUI.

## 5. Conclusions

This paper presents theoretical and practical insights regarding citizen involvement in research on Problematic Usage of the Internet (PUI), and demonstrates the potential to enrich this area of public mental health. Specifically, we have proposed: (a) a conceptual framework for the early involvement of citizens, at the planning stage of an emerging research; and (b) a template for initial consultation with citizens on PUI. Special emphasis has been placed on the ethical considerations for citizen involvement in PUI. Our work has been informed by the community case studies, performed in six European countries and coordinated by a group of experienced researchers from the European Network on PUI, who worked alongside diverse groups of interested citizens (students, parents, teachers, and health professionals). The participating researchers were national representatives of countries with limited experience in PPI and without clear national standards for citizen involvement in science. The experts working in future research settings alongside their wider academic and citizen communities, could greatly benefit from this shared learning and practical experience.

The proposed ethical aspects for citizen consultation on PUI are a novel contribution in this field. In our opinion, they provide a valuable foundation on which to advance our understanding and generate international strategies for citizen involvement in PUI. We hope that other studies will follow, producing a more balanced, inclusive and comprehensive outlook on the perspective of citizens regarding research in PUI, and enabling voices of citizens to be heard, valued and acted upon.

## Data Availability Statement

The original contributions presented in the study are included in the article/supplementary material, further inquiries can be directed to the corresponding author/s.

## Ethics Statement

Ethical review and approval was not required for the study on human participants in accordance with the local legislation and institutional requirements. Written informed consent for participation was not required for this study in accordance with the national legislation and the institutional requirements.

## Author Contributions

BG, CS, AV, and JJ contributed to the conceptualization and design of the study. PB, KF, AF-N, LI, ZK, DM, and MV-R conducted consultations with citizens. BG collated information on citizen consultation exercises and drafted the study. NH and JJ proofread the study. AF-N covered the costs for additional editing by specialized proofreading services. CS and JJ supervised the study. All authors contributed to the writing, revision of the study, and approved the final manuscript.

## Conflict of Interest

The authors declare that the research was conducted in the absence of any commercial or financial relationships that could be construed as a potential conflict of interest.
